# Dynamical Modeling of the Core Gene Network Controlling Flowering Suggests Cumulative Activation From the *FLOWERING LOCUS T* Gene Homologs in Chickpea

**DOI:** 10.3389/fgene.2018.00547

**Published:** 2018-11-20

**Authors:** Vitaly V. Gursky, Konstantin N. Kozlov, Sergey V. Nuzhdin, Maria G. Samsonova

**Affiliations:** ^1^Theoretical Department, Ioffe Institute, Saint Petersburg, Russia; ^2^Systems Biology and Bioinformatics Laboratory, Peter the Great Saint Petersburg Polytechnic University, Saint Petersburg, Russia; ^3^Molecular and Computational Biology, University of Southern California, Los Angeles, CA, United States

**Keywords:** chickpea, flowering time, FT genes, ICCV 96029, CDC Frontier, dynamical model

## Abstract

Initiation of flowering moves plants from vegetative to reproductive development. The time when this transition happens (flowering time), an important indicator of productivity, depends on both endogenous and environmental factors. The core genetic regulatory network canalizing the flowering signals to the decision to flower has been studied extensively in the model plant *Arabidopsis thaliana* and has been shown to preserve its main regulatory blocks in other species. It integrates activation from the *FLOWERING LOCUS T* (*FT*) gene or its homologs to the flowering decision expressed as high expression of the meristem identity genes, including *AP1*. We elaborated a dynamical model of this flowering gene regulatory network and applied it to the previously published expression data from two cultivars of domesticated chickpea (*Cicer arietinum*), obtained for two photoperiod durations. Due to a large number of free parameters in the model, we used an ensemble approach analyzing the model solutions at many parameter sets that provide equally good fit to data. Testing several alternative hypotheses about regulatory roles of the five *FT* homologs present in chickpea revealed no preference in segregating individual *FT* copies as singled-out activators with their own regulatory parameters, thus favoring the hypothesis that the five genes possess similar regulatory properties and provide cumulative activation in the network. The analysis reveals that different levels of activation from *AP1* can explain a small difference observed in the expression of the two homologs of the repressor gene *TFL1*. Finally, the model predicts highly reduced activation between *LFY* and *AP1*, thus suggesting that this regulatory block is not conserved in chickpea and needs other mechanisms. Overall, this study provides the first attempt to quantitatively test the flowering time gene network in chickpea based on data-driven modeling.

## Introduction

The depleted genetic diversity of many domesticated agriculturally important plants is a common problem for breeders, providing an obstacle in developing new forms with desired features. One such feature important for domesticated chickpea (*Cicer arietinum*) is early flowering time, which enforces more rapid transition from vegetative to reproductive growth. Due to high sensitivity of chickpea to ascochyta blight, it is essential to reduce the full plant cycle, from sowing to maturation, in order to fit it to relatively short growing seasons having dry weather and, hence, low disease pressure (Kumar and Abbo, 2001). These growing seasons are quite short in major chickpea growing regions, pushing breeders to developing chickpea lines with early flowering time. Thus, it is important to identify key genes regulating floral transition and quantitatively understand the behavior of the flowering time gene network.

The floral transition has been intensively studied in model organisms, such as Arabidopsis (*Arabidopsis thaliana*) (Srikanth and Schmid, 2011; Andrés and Coupland, 2012), and in other plants, including important crops and legumes (Kumar and Abbo, 2001; Dong et al., 2012; Shrestha et al., 2014; Blümel et al., 2015; Peng et al., 2015; Weller and Ortega, 2015; Zhang et al., 2016; Ridge et al., 2017). Flowering starts in response to various environmental signals, including photoperiod and vernalization, and endogenous signals, such as autonomous and circadian clock, and molecular pathways have been identified conducting these signals to the core gene network that integrates them into a binary decision to flower. Despite the high complexity of these pathways and many unknown regulators, it has been shown that key genes regulating the process are conserved between species. In particular, the flowering signals lead to the elevated expression of the floral pathway integrator gene *FLOWERING LOCUS T* (*FT*), or its homologs, in the leaves (Kardailsky et al., 1999; Kobayashi et al., 1999; Pin and Nilsson, 2012; Jaeger et al., 2013).

**Figure 1:**
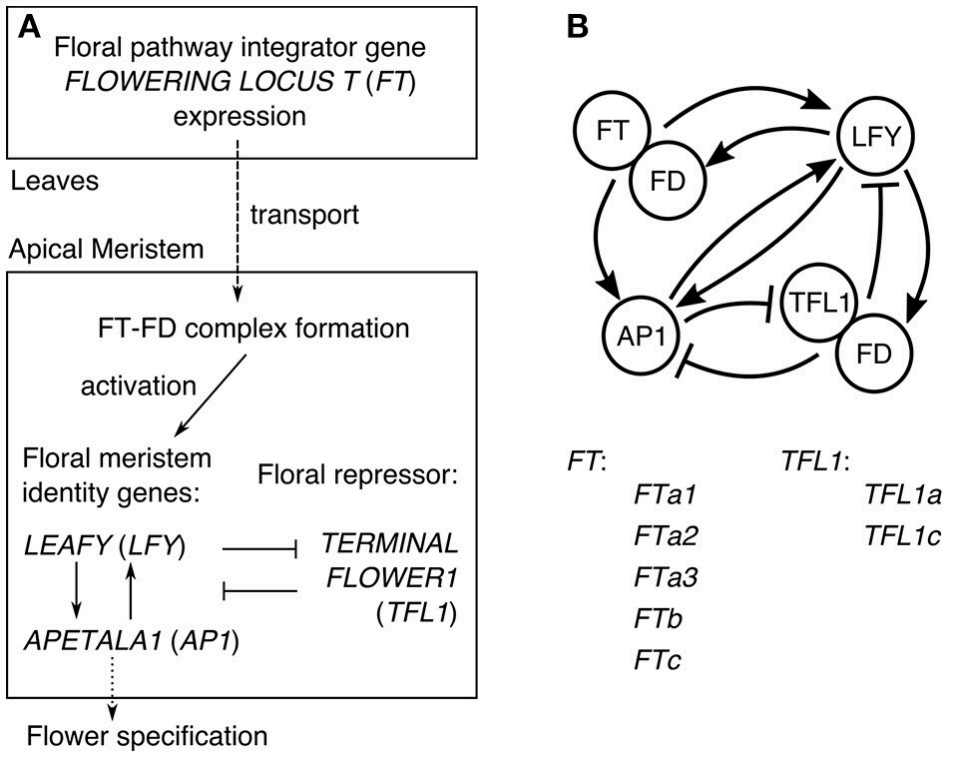
The core gene network controlling floral transition. **(A)** The general scheme of processes involved in floral transition. **(B)** The graph of the regulatory interactions proposed for Arabidopsis, and the list of the *FT* and *TFL1* homologs in chickpea considered in our model. The interaction graph was adopted from (Jaeger et al., 2013).

In Arabidopsis, the understanding of the core gene network integrating the flowering signals transmitted via the expression of *FT* has evolved to the general scheme illustrated in Figure 1A (Jaeger et al., 2013). FT is a mobile factor transported from the leaves to the apical meristem, where it forms the complex with the transcription factor FD. This complex activates the meristem identity genes *LEAFY* (*LFY*) and *APETALA1* (*AP1*), which also activate each other. The expression of *AP1* activates genes controlling flower development and thus can be considered as the output of the network specifying the floral transition (Kaufmann et al., 2010). In order to keep the center of the shoot apical meristem in a vegetative state, the key floral repressor *TERMINAL FLOWER1* (*TFL1*) inhibits expression of *LFY* and *AP1* in this region. The resulting gene interaction graph takes the form shown in Figure 1B, incorporating evidence for some additional interactions: TFL1 acts as a repressor in the complex with FD, LFY activates *FD*, and AP1 represses *TFL1*. As many genes are omitted, each node in the graph in fact represents a group of genes (Jaeger et al., 2013).

The knowledge about the regulatory interactions between the genes from Figure 1 has been obtained via extensive genetic studies, and it provides a unique opportunity for computational modeling of this gene regulatory network, when experimental data on the system behavior is available. The modeling allows to gain mechanistic insights into specific properties of the floral transition system and produce testable predictions. Jaeger et al. (2013) elaborated a dynamical model of the core network from Figure 1 based on the data on the flowering time for a set of the wild type and mutant Arabidopsis genotypes. They showed that the floral transition dynamics can be explained by splitting the network into several feedback and forward loops, each bearing a clear functional role (Pullen et al., 2013). Leal Valentim et al. (2015) studied a similar gene network, particularly considering that the complex TF-FD activates *LFY* via the intermediate transcription factors SOC1 and AGL24. They measured expression dynamics of all genes involved and used this data to calibrate a dynamical model. Using this data-driven approach, they tested various hypotheses about regulation of *LFY* by SOC1 and AGL24 and showed that perturbations can spread through the network in a nonlinear way.

A possibility to extend these results to chickpea depends on what we know about the inflorescence genes in this species. We concentrate on two chickpea cultivars in this study, CDC Frontier and ICCV 96029. CDC Frontier is a photoperiod-sensitive kabuli chickpea cultivar developed at the University of Saskatchewan (Warkentin et al., 2005), exhibiting relatively late flowering (Daba et al., 2016; Ridge et al., 2017). The reference genome sequence was obtained for this cultivar (Varshney et al., 2013). ICCV 96029 is a photoperiod-insensitive desi chickpea cultivar developed by the International Crops Research Institute for the Semi-Arid Tropics, India, representing the earliest flowering chickpea cultivar currently known. Quantitative trait loci associated with early flowering were investigated, and it was shown that a single recessive allele with some additional modifiers confer early flowering of ICCV 96029 (Kumar and van Rheenen, 2000; Gaur et al., 2015; Upadhyaya et al., 2015; Mallikarjuna et al., 2017). Ridge et al. (2017) provided evidence that a mutation in an ortholog of the key circadian gene *ELF3* can be associated with earliness in ICCV 96029 under short day growth conditions, but their analysis of the expression of clock genes in ICCV 96029 did not reveal any clear differences for this cultivar.

In contrast to the single *FT* gene in Arabidopsis, Ridge et al. (2017) identified five *FT* homologs in chickpea: *FTa1, FTa2, FTa3, FTb*, and *FTc*, named according to affiliation with one of the three clades (*FTa, FTb*, and *FTc*). They also found two chickpea orthologs of *TFL1* (*TFL1a* and *TFL1c*). Furthermore, Ridge et al. (2017) measured the expression dynamics of the homologs of all genes from the core gene network for CDC Frontier and ICCV 96029 under two growth conditions (short day, SD, and long day, LD) and identified specific differences in expression between these genotypes. In particular, they noted that the up-regulation of *FT* and *AP1* expression was synchronous with floral bud initiation, thus confirming that regulation of floral transition in chickpea occurs via the *FT* gene family.

We aimed to investigate a possibility to extend the core gene network from Figure 1 to chickpea. Assuming this network is conserved, we developed a dynamical model of gene expression and applied it to the previously published expression time series (Ridge et al., 2017). We used the resultant model to dissect interactions in which targets were found insensitive to regulator action. This points to chickpea specific deviations in regulation of floral transition. We also studied if the *TFL1* homologs are mutually distinguishable in the context of the model. Finally, we tested several hypotheses about how the *FT*-like genes combine in their activation of the meristem identity genes.

## Results

### Model

We modeled the flowering time gene network shown in Figure 1. We formulated the model in terms of the ordinary differential equations in which the change rates of gene product concentrations are regulated by the activators and inhibitors via the Hill-type regulation functions (the model equations (1–5) are described in details in section Materials and Methods). The formulation of the model equations depends on how we combine the activation from the *FT*-like genes. The baseline model (model, or hypothesis, *H0*) assumes that the five *FT* homologs are mutually indistinguishable in their activation of the meristem identity genes (*LFY* and *AP1*). In this model, FD forms the complex with the total FT concentration equal to the sum of the protein concentrations from each *FT* homolog. The activation of *LFY* by the FT-FD complex is characterized in the model equations by the regulation function containing the following regulatory parameters: one Michaelis–Menten constant (*K*_8_), one Hill parameter (*n*_8_), and one maximal synthesis rate (*v*_8_) (see equation (6) in section Materials and Methods), and a similar set of regulatory parameters quantify the activation of *AP1* by the total FT concentration. An alternative model (*H1*) assumes that only one of the five FT's is enough to activate transition to flowering, so the concentration of only that FT participates in the complex FT-FD and activates *LFY* and *AP1* (see equation (7) in section Materials and Methods for the case of *LFY* activation). In another alternative model (*H2*), we tried to distinguish a single *FT* gene from the other four assuming that this singled-out gene has the regulatory parameters distinct from the rest of the *FT* genes, while these FT's still activate cumulatively (like in model *H0*). The activation from the singled-out *FT* gene and the activation from the total concentration of the rest of the *FT* genes are represented in the model by two distinct regulation functions (see equation (8) in section Materials and Methods for the case of *LFY* activation). Models *H1* and *H2* have five possible versions, where each version is associated with one *FT* homolog separated from the other *FT*-like genes. We tested only four of them, excluding *FTa3* from the analysis due to its very low expression in both growth conditions.

We applied the models to describe the previously published dynamic expression data for all genes from the core network measured in two chickpea cultivars, ICCV 96029 and CDC Frontier (Ridge et al., 2017). We failed to find a good model solution for the expression data from CDC Frontier (the best solution is shown in Supplementary Figure 1; we also discuss possible reasons in Discussion). Therefore, the rest of the paper describes modeling results for ICCV 96029.

### Parameter estimation and model solutions for ICCV 96029

Models *H0* and *H1* have the same number of free parameters (*k* = 31), and model *H2* has six parameters more (*k* = 37). We estimated values of these parameters by minimizing the weighted sum of squared residuals quantifying the difference between the model solution and the ICCV 96029 data for the two growth conditions (SD and LD) simultaneously (section Materials and Methods). The data comprised expression levels of five genes (*TFL1a, TFL1c, FD, LFY*, and *AP1*) in ICCV 96029 on 7 days under SD and LD, with the total number of data points equal to *m* = 70. After estimating the parameter values, we applied the Akaike information criterion corrected for small data samples for model comparison, as described further in the text.

As *k* was relatively large, we refused to estimate the parameter values by fitting the model to the data from one condition (either LD or SD) and testing on the data from the other condition. In that case, the number of parameters *k* in model *H2* would exceed the number of data points (*m* = 35 in LD or SD) and *k* in other model versions would be close to *m*, and that would complicate the application of the Akaike information criterion for model comparison. As a control, we performed the fitting to the LD data and tested on the SD data in model *H0* and made sure that the corresponding solutions were qualitatively similar to the two-conditions fitting results (Supplementary Figure 2).

We further circumvented an overfitting potential of the two-conditions fitting applying the ensemble approach in the analysis of model behavior (Samee et al., 2015). In this approach, all sets of parameter values and solutions resulted from the fitting procedure were considered as equally suited for biological conclusions, and the conclusions were derived based on the analysis of the whole ensemble of the solutions and optimized parameter values.

**Figure 2:**
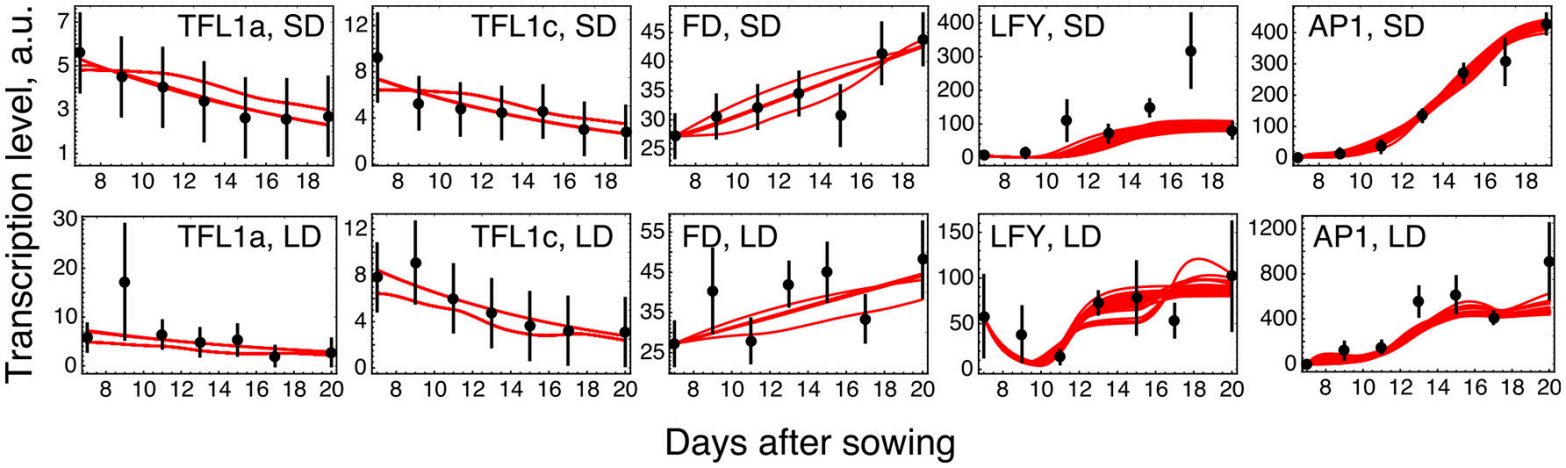
Model *H0* solutions for ICCV 96029 under two growing conditions. The model solutions (red curves) corresponding to all parameter sets found by optimization are shown for five flowering time genes and for the short day (SD, upper panels) and long day (LD, lower panels) conditions. The black dots and error ranges are the mean expression data and standard deviation, respectively, taken from (Ridge et al., 2017).

The parameter optimization under hypothesis *H0* resulted in the model solutions of very similar quality (Figure 2; distributions of the estimated parameter values are shown in Supplementary Figure 3). The model correctly reproduces the main characteristics of the data. The dynamic increase of LFY and AP1 concentrations can be explained by activation from the rising expression of the *FT* genes. LFY activates *FD*, resulting in the dynamic increase of its expression. Finally, the floral repressors TFL1a and TFL1c decrease in time due to repression by AP1.

### Reduced *LFY* and *AP1* activation

**Figure 3:**
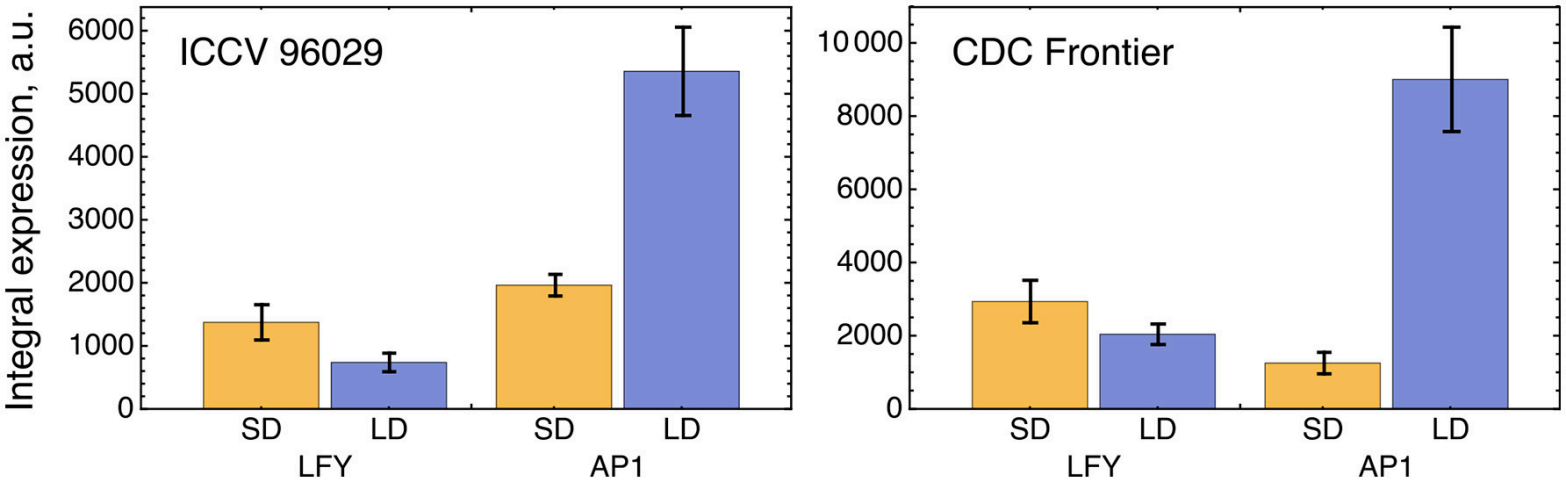
Integral expression levels of *LFY* and *AP1* under two growth conditions in two cultivars, based on the data from (Ridge et al., 2017). At each time where data was available, 100 expression values were sampled from the normal distribution with the mean and s.d. presented at this temporal point in the data. These values then were interpolated across time, producing a set of 100 expression dynamics, and these dynamics were integrated over time. The chart and error bars show means and standard deviations, respectively, over this set of the integral values.

The solution in Figure 2 shows somewhat insufficient expression levels of both *LFY* under SD and *AP1* under LD. The analysis of the expression data reveals that LFY behaves rather counterintuitively under SD as compared with LD and differs in this behavior from AP1. Namely, *LFY* is down-regulated in LD compared to SD, despite the increased activation from the raising expression of the *FT* genes in LD compared to SD, and this holds both for ICCV 96029 and CDC Frontier (Figure 3). In contrast, the integral expression of *AP1* increases from SD to LD in accordance with the rising activation from FT. This anticorrelation between LFY and its sole activators (FT and AP1) observed in the data hampers the model in finding a better solution.

**Figure 4:**
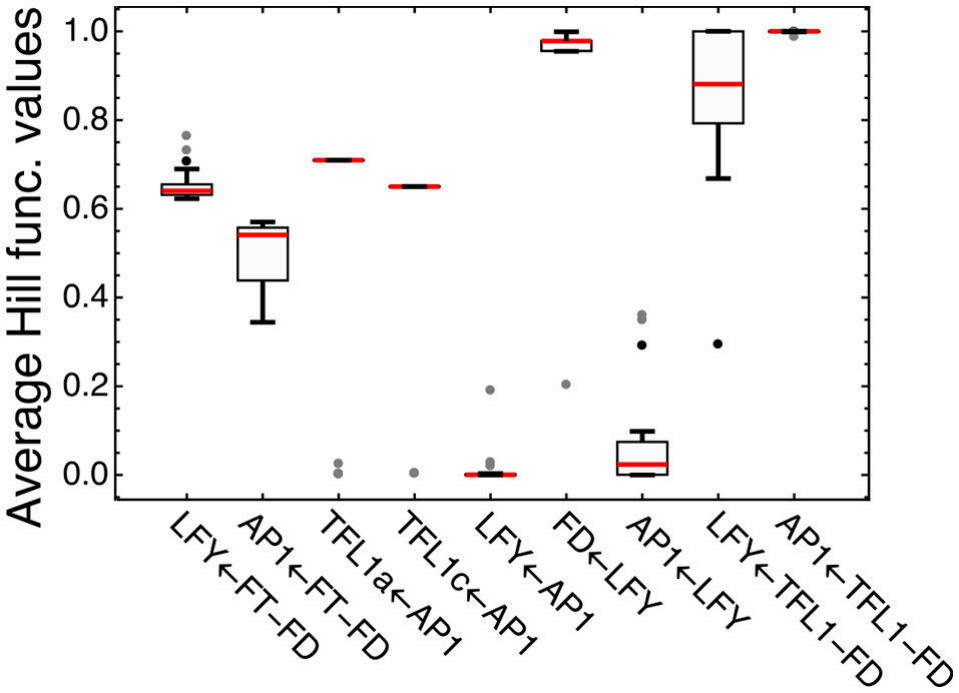
Average values of the regulation functions characterizing regulation in the model for ICCV 96029. For each set of the optimized parameter values, the averaged values of the regulation functions from the model equations were obtained by integrating these functions over time under SD and LD and dividing by the integration time interval; the figure shows box plots of the distributions of these values over all sets of the optimized parameter values. The type of regulation corresponding to each regulation function is shown on the horizontal axis, where arrow indicates the direction of the regulation. FT-FD and TFL1-FD denote the complexes of FD with all FT and two TFL1 proteins, respectively. Dots show outliers.

We analyzed how LFY and other transcription factors are involved in their regulations in the model for ICCV 96029 by plotting average values of the Hill functions which implement in the model equations each regulatory interaction from the gene network (Figure 4). An active regulation tends to keep the Hill function value between 0 and 1, while the limit values (0 or 1) evidence that the interaction between genes is saturated, with no sensitivity to specific expression levels of the regulators. This type of saturation occurs for activation of *LFY* by AP1, with the corresponding Hill function values pushed to zero. Activation of *AP1* by LFY is also characterized by the Hill function values close to zero, but the analysis of the Jacobian values of the right-hand side of the model equations for this regulation still shows relatively high LFY influence on *AP1* (Supplementary Figure 4). Another saturated regulation involving LFY is activation of *FD*. At the same time, *LFY* is sensitive to its repressors (the complexes TFL1a-FD and TFL1c-FD), in contrast to the saturated repression of *AP1* by these complexes (Figure 4). Overall, this analysis of the model and expression data suggests that there are regulators of *LFY* missing in the core gene network under study.

Figure 4 shows four regulations characterized by the average Hill function values that are considerably far from the saturation limits: activation of *LFY* and *AP1* by FT and repression of *TFL1a* and *TFL1c* by AP1. This fact allows us to use the model for testing various alternative hypotheses about these regulations.

### Difference in *TFL1a* and *TFL1c* expression can be explained by different regulation by AP1

**Figure 5:**
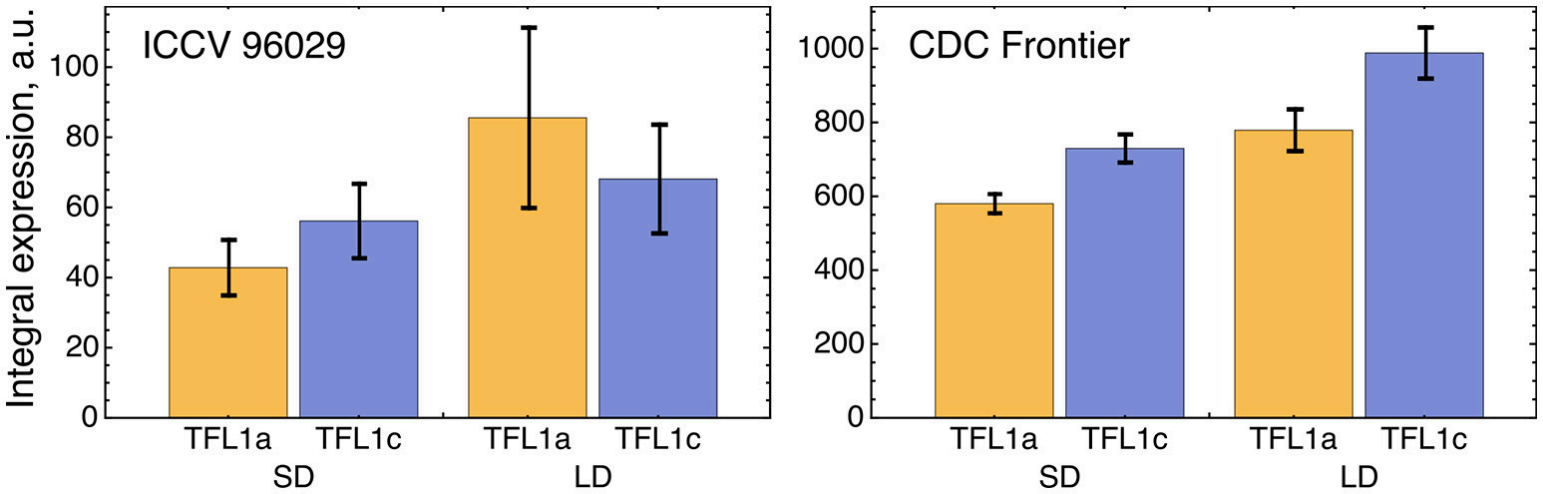
Integral expression levels of *TFL1a* and *TFL1c* under two growth conditions in two cultivars, based on the data from (Ridge et al., 2017). The integral expression levels were calculated as described in Figure 3.

We tested a hypothesis that a small difference in *TFL1a* and *TFL1c* expression observed in the data (Figure 5) can be explained by different regulation by AP1. Because of this difference in the expression, we included TFL1a and TFL1c in the model as two distinct dynamical variables whose dynamics are under control of the following four parameters per factor (equations (1–2) in section Materials and Methods): maximal expression rate *v*_*i*_, dissociation constant *K*_*i*_, cooperativity parameter *n*_*i*_, and degradation rate λ_*i*_ (*i* = *1,2*). If the model fitting produced no significant difference in these parameters between TFL1a and TFL1c, there would be no means to distinguish between these factors in the model and we would have to consider a single dynamical variable TFL1 = TFL1a + TFL1c instead. If the difference in parameter values exists, there is an interesting question about whether this difference can be explained by different regulation from AP1. If AP1 is indeed involved, a statistically significant difference should exist between values of the regulatory parameters *K*_1_ and *K*_2_ and/or between values of *n*_1_ and *n*_2_, because these parameters are associated with repression of *TFL1a* and *TFL1c* by AP1. A possible difference in *v*_*i*_ and/or λ_*i*_ should be attributed to other, AP1 independent, factors.

The optimized parameter values for TFL1a and TFL1c form two clearly separated clusters, which correspond to the main box (“main cluster”) and the outliers (“outlying cluster”) in the AP1→TFL1a and AP1→TFL1c parts of Figure 4, and it is already seen in this figure that the regulation by AP1 differs between the analyzed target genes within the main cluster. The Hill exponents *n*_*i*_ are the same in the main cluster for both TFL1a and TFL1c (*n*_*i*_ = 1, *i* = 1,2), but we see the significant difference in *K*_*i*_ values in this cluster: *K*_1_ = 561.14 ± 0.13 (TFL1a) and *K*_2_ = 401.14 ± 0.08 (TFL1c) (*p*-value = 2 × 10^−9^). Therefore, the model suggests different regulatory properties of AP1 in its action on the genes *TFL1a* and *TFL1c*, linked to possible different association kinetics to their promoters. The outlying cluster is characterized by a small influence of AP1 and contain only from 5 to 6 parameter sets with very similar *K*_*i*_ and *n*_*i*_ values, so we consider this cluster as not relevant.

### Model suggests cumulative activation by the *FT* homologs

**Figure 6:**
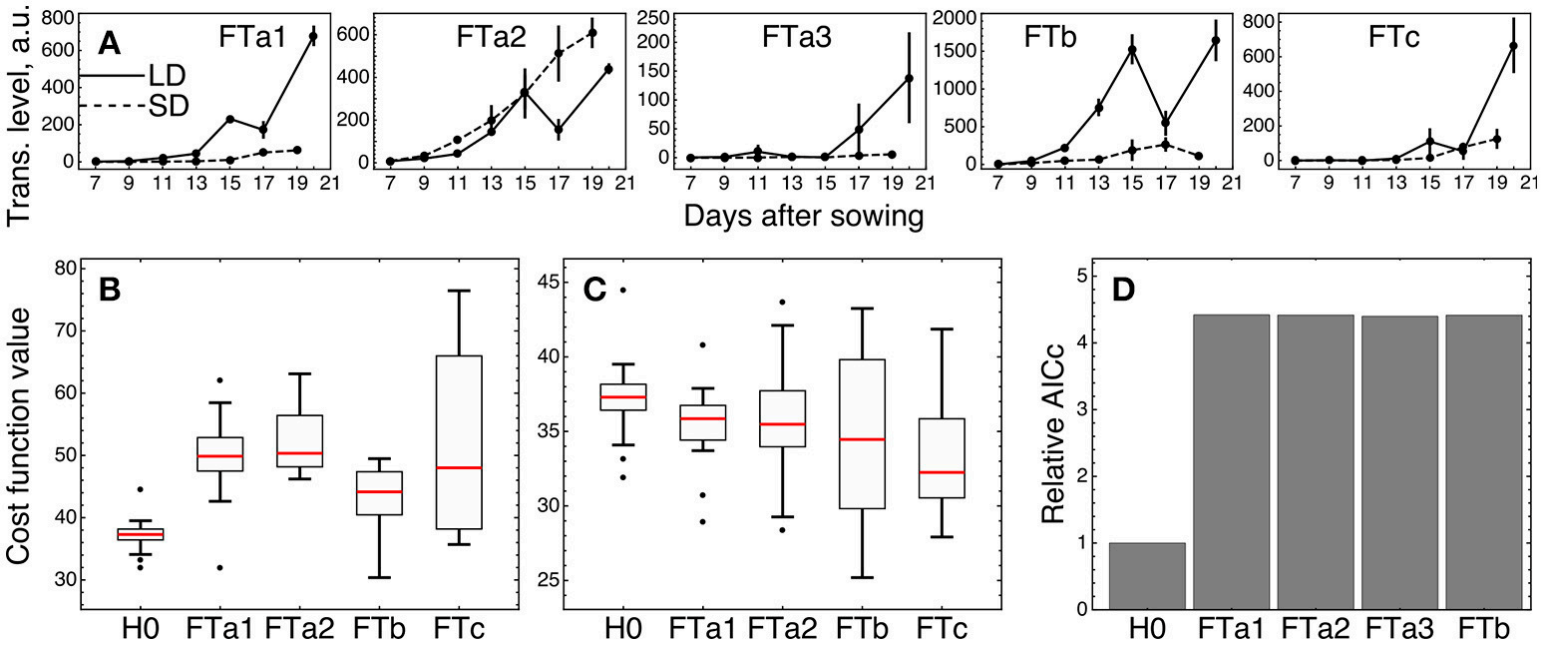
Testing alternative hypotheses on regulation by the *FT* genes in ICCV 96029. **(A)** Expression data of the *FT* genes in ICCV 96029 under SD and LD; reproduced from (Ridge et al., 2017). **(B)** Values of the cost function (weighted residual sum of squares; equation (9) in Materials and Methods) quantifying the goodness of fit for model *H0* and four versions of model *H1*, for all optimized parameter sets. Each version of model *H1* is marked on the bottom of the panel by the name of the *FT* gene participating as a sole FT activator in the model. **(C)** The same as in **(B)**, but for model *H2*. Each version of model *H2* is marked on the bottom of the panel by the name of the *FT* gene singled out in the model equations from the other *FT* genes. **(D)** Akaike information criterion corrected for small data samples (AICc; equation (10) in Material and Methods) for *H0* and four versions of model *H2*, marked as in **(C)**. The relative values of AICc normalized to the *H0* value are shown. The use of a more conventional form of AICc yields a similar figure (Supplementary Figure 8 and Supplementary Text).

We tested whether an individual *FT* gene stands out against the other *FT* homologs by fitting the three versions of the model (models *H0, H1*, and *H2*) described above and in Materials and Methods, with subsequent comparison of their fitting quality. We considered only four of the five *FT* genes in the tests excluding *FTa3*, since its expression was small relative to the other ones (Figure 6A).

We first checked if a single *FT* gene can provide the full activation from the *FT* gene family in the network, thus serving as a unique transmitter of the flowering signal (model *H1*). Under this assumption, we replaced the sum of FT concentrations in the model equations by the concentration of one of the four FT's and fitted each resulted version of the model to the expression data for ICCV 96029. For each tested *FT* gene, model *H1* demonstrated worse fitting quality as compared to the baseline model with the cumulative activation from all *FT* genes (model *H0*) (Figure 6B; *p*-value = 3 × 10^−7^ for FTa1 as the sole activator; 7 × 10^−9^, FTa2; 2 × 10^−5^, FTb; 10^−4^, FTc). Breaking the cost function into the separate SD- and LD-related components reveals that all versions of model *H1* have worse quality in description of the LD data and all except the *FTa2*- and *FTc1*-related models have worse description of the SD data (Supplementary Figure 5). Since models *H0* and *H1* have the same number of parameters, neither of them is prone to overfitting to a larger extent than the other one, and, hence, we can conclude about better relevance of model *H0* based on the fitting quality comparison and without applying additional quality measures.

As several *FT* genes are required for better description of the expression data, a question yet remains about whether different FT's activate the meristem identity genes differently in terms of their regulatory parameters. We implemented this possibility in model *H2* by singling an FT out from the other four and adding a new regulation function to the model equations representing the activating action of this FT with its own regulatory parameters (*v, K*, and *n*), while preserving in the equations the activation from the sum of the other FT concentrations. Model *H2* exhibited a better fitting quality than *H0* for the singled-out genes *FTa1* (*p*-value = 0.005) and *FTc* (*p*-value = 0.0004), with no improvement for the other two *FT* genes (*p*-value = 0.09 for the singled-out *FTa2* and 0.12 for *FTb*) (Figure 6C). Both *FTa1*- and *FTc*-related models *H2* demonstrate better fit to the LD-data, with no significant improvements in fits to the SD-data (Supplementary Figure 6).

We can try to find features in the expression of *FTa2* and *FTb* that can be attributed to their worse individual performance in the model. Figure 6A shows that the expression dynamics of *FTa2* is almost identical under SD and LD for a long time and becomes down-regulated under LD at later days, in contrary to the behavior of all other FT's and to the up-regulation of *AP1* in LD (Figure 3). At the other extreme, the up-regulation of *FTb* in LD is the strongest among the *FT* genes, and this raise in expression might be too large to represent the difference between SD and LD adequately. However, model *H1* with FTb as the only FT activator performs best among all *FT* genes on average (Figure 6B), and both *FTb*-related models (*H1* and *H2*) provide the lowest cost function values among all models, including *H0* (see the minimal cost values in Figures 6B,C), which hints at possible importance of this gene.

The observed better performance of models *H2* with the singled-out genes *FTa1* and *FTc* can be related to overfitting, since model *H2* has six parameters more than the baseline model *H0*. We controlled this by evaluating the Akaike information criterion corrected for small data samples (AICc; equation (10) in section Materials and Methods), which assesses the quality of a model applied to a data by combining the fitting quality of the model and its complexity in terms of the number of free parameters. Smaller values of this measure correspond to better models. AICc evaluation reveals that its value for each version of model *H2* is more than four times larger than for model *H0* (Figure 6D), which suggests that the complexity added to model *H2* is not justified by the resulted improvement in fitting. Therefore, we conclude that the model with the cumulative activation from all *FT* genes (model *H0*) is the most relevant for the given expression data.

## Discussion

We presented a computational model of the core gene network controlling the floral transition and investigated its ability to describe the expression data in two chickpea cultivars. We were able to find good model solutions for ICCV 96029, which suggests a general conservation of the core gene network from Figure 1 in this chickpea cultivar. On the other hand, the modeling results were negative for CDC Frontier. A possible reason for this could be related to the specific choice of the modeling formalism. This explanation does not seem likely, since the modeling formalism is quite general and has been successfully applied to the same gene network in Arabidopsis (Leal Valentim et al., 2015). Another explanation which we find more probable is that this gene network is more perturbed in CDC Frontier than in ICCV 96029.

Several key differences between CDC Frontier and ICCV 96029 were reported based on the analysis of the expression data (Ridge et al., 2017): ICCV 96029 exhibits much earlier and much stronger up-regulation of the expression of *AP1*, according to the earlier appearance of visible floral buds as compared to CDC Frontier. The floral repressors *TFL1a* and *TFL1c* have lower expression levels in ICCV 96029 than in CDC Frontier, also in accordance with the early flowering of the former. On the other hand, the differences in expression of *FD* and *LFY* are not as visible between the cultivars.

**Figure 7:**
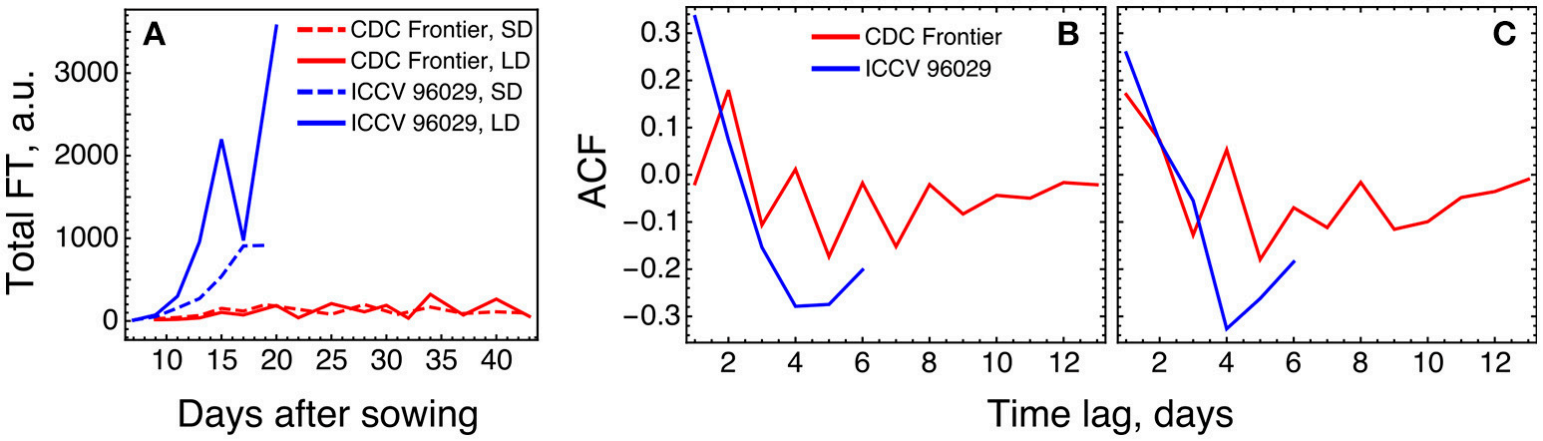
Difference in FT behavior between ICCV 96029 and CDC Frontier, based on the expression data from (Ridge et al., 2017). **(A)** The dynamics of the sum of concentrations of all five FT transcripts, for the two cultivars and two growth conditions. Developing floral buds were first detected at 15 days (under SD) and 13 days (LD) in ICCV 96029 and at 31 days (SD) and 32 days (LD) in CDC Frontier (Ridge et al., 2017). **(B)** Autocorrelation function (ACF) for the expression data time series of the *FT* genes. ACF estimates similarity (correlation) between data points as a function of the time lag between them. For each time lag value, an ACF value was calculated for the expression time series for each *FT* gene and growth condition (SD and LD), and then an average ACF was calculated over the *FT* genes and conditions. **(C)** The same as in **(B)** but for the expression dynamics of the genes *TFL1a, TFL1b, FD, LFY*, and *AP1*.

The expression levels of the *FT* genes in the data are significantly different for the two cultivars, and the total FT concentration in CDC Frontier can be estimated as close to the background levels (Figure 7A). This can partially explain why the model is not feasible for the expression data from CDC Frontier. Such small FT levels could possibly be related to the observed fact that the first floral buds, appeared in CDC Frontier at 31 days after sowing in SD and at 32 days in LD, were abortive, although the low expression of some of these genes persisted for much longer time (Ridge et al., 2017). Furthermore, investigation of the autocorrelation functions of the *FT* expression time series reveals very different patterns in the FT signals between the cultivars (Figure 7B), and these patterns are translated to the rest of the core network genes almost without changes (Figure 7C). It is interesting to note a periodic signal in the FT dynamics in CDC Frontier with a period of two days, although this signal can yet be an experimental artifact related to low expression levels.

Another important difference between the cultivars that we see in the data and that might contribute to the difference in the modeling results concerns the dependence between concentrations of TFL1a/TFL1c and LFY/AP1. TFL1a and TFL1c repress *LFY* and *AP1*, and AP1 represses the *TFL1*-like genes (Ratcliffe et al., 1999; Kaufmann et al., 2010). Therefore, we should expect that these two groups of transcripts should avoid coexistence in the data and, hence, exhibit a negative correlation over time. We do see this correlation in the data from ICCV 96029, but not from CDC Frontier (Table 1). Moreover, Table 1 shows that these mutual repressors tend to show a positive correlation in the CDC Frontier data. Regardless of whether this inconsistency in the CDC Frontier data should be attributed to an artifact or it hints at alternative regulations between the *TFL1*-like genes and the inflorescence identity genes in this cultivar, this property evidently impedes the modeling success under given assumptions.

**Table 1:** Correlations between the expression dynamics of TFL1a/TFL1c and LFY/AP1 in the data from (Ridge et al., 2017).

	**ICCV 96029**		**CDC Frontier**	
	**SD**	**LD**	**SD**	**LD**
TFL1a vs. LFY	−0.89 (*P* < 0.01)*	−0.57 (*P* = 0.10)	0.80 (*P* < 0.01)*	0.14 (*P* = 0.36)
TFL1a vs. AP1	−0.89 (*P* = 0.01)*	−0.64 (*P* = 0.07)	0.18 (*P* = 0.31)	−0.14 (*P* = 0.41)
TFL1c vs. LFY	−0.61 (*P* = 0.10)	−0.64 (*P* < 0.01)*	0.81 (*P* < 0.01)*	0.33 (*P* = 0.13)
TFL1c vs. AP1	−0.96 (*P* < 0.01)*	−0.86 (*P* < 0.01)*	0.22 (*P* = 0.20)	0.13 (*P* = 0.28)

*The Spearman rank correlation coefficient ρ was calculated for each cultivar (CDC Frontier and ICCV 96029) and growth condition (SD and LD). The p-values (P) were calculated by one-tailed permutation test, and the p-values below 0.05 are marked with asterisk*.

It has been shown that *LFY* is involved in positive regulation of *AP1* and is positively regulated by AP1 in Arabidopsis (Wagner et al., 1999; Jaeger et al., 2013; Leal Valentim et al., 2015). Our modeling results suggest that some additional factors should exist providing insufficient activation of these genes in the model for chickpea. The counterintuitive increase in the integral expression of *LFY* under SD as compared with LD, contrary to the decreasing activation from the *FT*-like genes, may indicate that additional activators of *LFY* participate under SD and compensate the missing activation. We believe that the absence of such factors in the core gene network considered in our model and, as a consequence, the inability to properly handle the LD vs. SD changes in expression is the reason why AP1 is almost excluded as an activator of *LFY* in the model solutions. In other words, this allows for the hypothesis that the *LFY*-*AP1* regulation module is not conserved in chickpea. However, we should also consider the possibility that the LD vs. SD increase in expression of *LFY* is due to insufficient quality of the data. Future work, both modeling and experimental, should clarify this point.

Since ICCV 96029 is day length neutral and floral transition is conferred via the *FT* genes, we might expect no difference in *FT* expression between SD and LD treatments in this cultivar. However, the expression data by Ridge et al. (2017) shows an essential difference in expression of these genes (Figures 6, **7A**), and it is important that this difference is transferred to the SD/LD difference in expression of *AP1* (Figure 3), so that the key gene specifying flower meristem identity exhibits sensitivity to photoperiod according to the data. This expression data was collected from the plants with first visible floral buds appeared at 15 days after sowing in SD and 13 days in LD (Ridge et al., 2017), thus providing the two days difference in floral bud initiation time between SD and LD. This two days difference diverges from previous measurements showing no difference in this time in ICCV 96029 (19 days from seeding ± 0.0) (Daba et al., 2016), but it qualitatively matches with the observed difference in expression.

Irrespective of whether this match is confident or not, the observed raise in expression of the *FT* genes and *AP1* in LD suggests that some compensatory mechanisms, or missing repressors, should exist diminishing the influence of that extra expression on the time to flower. It is reasonable to presume that these mechanisms should operate in the post-inductive phase of flower development, as they take the increased expression of floral meristem identity genes as the input. However, this conjecture is not in correspondence with the previously observed fact that ICCV 96029 does not exhibit photoperiod sensitivity on any of the pre-, inductive, or post-inductive phases of flower development (Daba et al., 2016). We believe this expression-based photoperiod sensitivity effect in ICCV 96029 is a fascinating subject for further studies.

An important difference of legumes and other species from Arabidopsis is in multiple orthologs of the inflorescence genes, such as *FT*, that present in a single copy in Arabidopsis (Pin and Nilsson, 2012). The regulatory roles of individual copies can sometimes be separated from the others; for example, *FTb* has been shown to have the leading role in pea (Hecht et al., 2011). The main purpose of our modeling approach was to infer possible differences in regulatory roles or other properties associated with the five *FT* homologs and two *TFL1* homologs in chickpea (Ridge et al., 2017). It is important that the model and expression data in principle allow to perform such inference, as the fitting results reveal that both *FT*- and *TFL1*-like genes are involved in active regulations.

AP1 was shown to repress *TFL1*-like genes (Liljegren et al., 1999; Kaufmann et al., 2010; Jaeger et al., 2013), and we found that this repression can be different for *TFL1a* and *TFL1c* in chickpea. As this difference concerns only the values of the equilibrium dissociation constant *K*, we can suggest that AP1 has different binding properties to the promoters of *TFL1a* and *TFL1c*.

Visual comparison between the expression of the five *FT*-like genes in ICCV 96029 does not help in differentiating their regulatory properties. Our modeling results support the cumulative activation model, in which all FT proteins have very similar regulatory properties and activation of the meristem identity genes occurs via the total FT concentration. Analyzing their expression data, Ridge et al. pointed at *FTb* as particularly important for induction of flowering (Ridge et al., 2017). However, this gene becomes indistinguishable from the others if we put it in the modeling context. The ensemble of model fits in which this gene is singled out does not improve the model, and we get the same conclusions using the Akaike information criterion to assess the relative performance of the model. On the other hand, we found that singling *FTb* out produced the lowest values of the minimal cost in all types of the computational experiments, suggesting that its potential of being the leading *FT* activator is not exhausted and is not seen only due to possible imperfections of the model and/or data.

As any modeling approach, our model has limitations. Perhaps the most important one concerns the large number of free parameters. We tackled this inevitable problem by utilizing the ensemble approach in the analysis of the model behavior (Samee et al., 2015). Despite the existing interdependence between the model parameters, the optimized parameter values led to the set of very similar solutions for ICCV 96029. We drew any conclusions only based on the average over the ensemble of the optimized parameter values, thus utilizing the “wisdom of the crowd” principle. We note that, for example, both the model with the single *FTb* and the model with the singled-out *FTb* provide the minimal costs among all alternative models, while they do not perform better on average. Even with the given number of free parameters, the model was not able to reproduce the expression data from CDC Frontier, which, in particular, indicates that we cannot fit any data. Therefore, we believe that the ensemble approach increases the confidence of our results.

## Materials and methods

### Model equations

We model the expression of *TFL1a, TFL1c, FD, LFY*, and *AP1* with the following set of differential equations:

(1) $$\frac{du_{TFL1a}}{dt}=v_{1}\frac{{K_{1}}^{n1}}{{K_{1}}^{n1}+{u_{AP1}}^{n1}}-\lambda_{1}u_{TFL1a}\;,$$

(2) $$\frac{du_{TFL1c}}{dt}=v_{2}\frac{{K_{2}}^{n2}}{{K_{2}}^{n2}+{u_{AP1}}^{n2}}-\lambda_{2}u_{TFL1c}\;,$$

(3) $$\frac{du_{FD}}{dt}=v_{3}\frac{{u_{LFY}}^{n3}}{{K_{3}}^{n3}+{u_{LFY}}^{n3}}-\lambda_{3}u_{FD}\;,$$

(4) $$\begin{array}{l} \frac{du_{LFY}}{dt}=\left(v_{4}\frac{{u_{AP\text{1}}}^{n4}}{{K_{4}}^{n4}+{u_{AP\text{1}}}^{n4}}+f_{FT\to LFY}(t)\right)\times \\ \;\left(\frac{{K_{5}}^{n5}}{{K_{5}}^{n5}+{\left[u_{FD}\left(u_{TFL1a}+u_{TFL1c}\right)\right]}^{n5}}\right)-\lambda_{4}u_{LFY}, \end{array}$$

(5) $$\begin{array}{l} \frac{du_{AP1}}{dt}=\left(v_{5}\frac{{u_{LFY}}^{n6}}{{K_{6}}^{n6}+{u_{LFY}}^{n6}}+f_{FT\to AP1}(t)\right)\times \\ \;\left(\frac{{K_{7}}^{n7}}{{K_{7}}^{n7}+{\left[u_{FD}\left(u_{TFL1a}+u_{TFL1c}\right)\right]}^{n7}}\right)-\lambda_{5}u_{AP1}, \end{array}$$

where *u*'s describe the protein concentrations, *v*_*i*_ are the maximal protein synthesis rates, *K*_*i*_ are the Michaelis–Menten constants (which can be seen as the equilibrium dissociation constants for the regulators binding the target gene promoters in the case of a direct transcriptional regulation), *ni* are the Hill constants (accounting for the cooperative effects), and λ_*i*_ are the protein degradation constants. We do not model translation explicitly, but instead assume that protein concentrations are proportional to mRNA concentrations for simplicity.

The specific form of the equations is chosen according to the regulatory graph in Figure 1 and can be read as follows. The last terms on the right-hand side of all the equations represent degradation of each protein. The first term on the right-hand side of equation (1) is the regulation function describing repression of *TFL1a* by AP1. The same regulation function but with different parameters describes repression of *TFL1c* by AP1 in equation (2). The first term on the right-hand side of equation (3) represents activation of *FD* by LFY. The first brackets in equation (4) contains the sum of the activating inputs to *LFY* expression from AP1 (the first term in the sum) and the FT homologs (the function *f*_*FT*→*LFY*_(*t*), described below). This input is multiplied by the regulation function in the second brackets of this equation, representing repression of *LFY* by the FD-TFL1 complex. This repression is represented under the assumption that TFL1a and TFL1c have equivalent regulatory properties, and the concentration of the complex is proportional to the product of the FD concentration (*u*_*FD*_) and the total concentration of TFL1a and TFL1c (*u*_*TFL*1*a*_+*u*_*TFL*1*c*_). The first brackets in equation (5) contains the sum of the activating inputs to *AP1* expression from LFY (the first term in the sum) and the FT homologs (the function *f*_*FT*→*AP*1_(*t*), described below). This input is multiplied by the regulation function in the second brackets of this equation, representing repression of *AP1* by the FD-TFL1 complex.

We test three alternative hypotheses (*H0, H1*, and *H2*) about functions *f*_*FT*→*LFY*_ and *f*_*FT*→*AP*1_. Under the null hypothesis *H0*, we assume regulatory equivalence of the five *FT* homologs, so the total concentration of all FT proteins forms the complex with FD and activate *LFY* and *AP1* with a single Michaelis–Menten constant and a single Hill constant, according to the following expression:

(6) $$H0:\;f_{FT\to LFY}(t)=v_{6}\frac{{\left[u_{FD}\sum_{i=1}^{5}u_{i}(t-\tau )\right]}^{n8}}{{K_{8}}^{n8}+{\left[u_{FD}\sum_{i=1}^{5}u_{i}(t-\tau )\right]}^{n8}}\;,$$

$$u_{1}=u_{FTa1},\;u_{2}=u_{FTa2},\;u_{3}=u_{FTa3},\;u_{4}=u_{FTb},\;u_{5}=u_{FTc}\;,$$

and a similar expression for the function *f*_*FT*→*AP*1_ with the *AP1*-related constants *v*_7_, *K*_9_, and *n*9. The FT concentrations in equation (6) are calculated with a time delay τ, which is taken to transport FT from the leaves to the apical meristem.

In the hypothesis *H1*, we assume that a single *FT* gene (with index *k*) is capable to fully represent the FT-mediated activation of *LFY* and *AP1*:

(7) $$H1:\;f_{FT\to LFY}(t)=v_{6}\frac{{\left[u_{FD}u_{k}(t-\tau )\right]}^{n8}}{{K_{8}}^{n8}+{\left[u_{FD}u_{k}(t-\tau )\right]}^{n8}}\;,$$

and a similar expression for the function *f*_*FT*→*AP*1_ with the same *u*_*k*_ and with the *AP1*-related constants *v*_7_, *K*_9_, and *n*9.

Under the hypothesis *H2*, we assume that a member *u*_*k*_ of the FT family is distinguishable from the rest four members of the family in terms of regulation of *LFY* and *AP1*, so that we can separate it into a distinct regulation function with its own regulatory constants as follows:

(8) $$\begin{array}{l} H2:f_{FT\to LFY}(t)=v_{6}\frac{{\left[u_{FD}\sum_{i\neq k}^{4}u_{i}(t-\tau )\right]}^{n8}}{{K_{8}}^{n8}+{\left[u_{FD}\sum_{i\neq k}^{4}u_{i}(t-\tau )\right]}^{n8}}\\ \;+v_{7}\frac{{\left[u_{FD}u_{k}(t-\tau )\right]}^{n9}}{{K_{9}}^{n9}+{\left[u_{FD}u_{k}(t-\tau )\right]}^{n9}}\;, \end{array}$$

and a similar expression for the function *f*_*FT*→*AP*1_ with the *AP1*-related constants *v*_8_, *v*_9_ *K*_10_, *K*_11_, *n*10, and *n*11. The first term in equation (8) describes the cumulative activation from four FT proteins distinct from the FT protein with index *k*, whose activating input is represented by the second term in this equation. Depending on which gene of the *FT* family is singled out in the described way, we have five possible forms of *f*_*FT*→*LFY*_ and *f*_*FT*→*AP*1_ to test under hypothesis *H2*.

We solved numerically equations (1–5) replacing the concentrations of all regulators in the right-hand side of the equations with their expression data values interpolated in time. This effectively splits the model into four independent parts which do not contain common parameters: single equations for TFL1a, TFL1c, and FD, and the system of two equations for LFY and AP1 sharing the common parameter τ. The initial conditions for all proteins except TFL1a and TFL1c were equal to the value of each transcript at the first available day from the expression data (Ridge et al., 2017). Setting the initial conditions for TFL1a and TFL1c in the same way led to undesirable artifacts in the solutions resulted from the fitting procedure (Supplementary Figure 7); therefore, the initial conditions for these proteins were set to zero at *t* = 0, and the functions in the right-hand side of the model equations were obtained by interpolating the data values back to zero concentrations at *t* = 0. Numerical solution was obtained using either the *ode23s* solver in Octave or the *NDSolve* function in Wolfram Mathematica.

### Parameter estimation

The model contains 31 free parameters (7 *v*_*i*_'s, 9 *K*_*i*_'s, 9 *ni*'s, 5 λ_*i*_'s, and τ) under hypothesis *H0* and in each version of the model under hypothesis *H1*, and there are six more parameters in *H2*. For the ICCV 96029 cultivar, the parameter values were found by minimizing the following weighted residual sum of squares (*wRSS*):

(9) $$wRSS=\sum_{g=1}^{5}\sum_{k=1}^{T}\frac{{\left(u_{g}\left(t_{k}\right)-u_{g}^{dat}\left(t_{k}\right)\right)}^{2}}{{\sigma_{g,k}}^{2}}\;,$$

in which the difference between the model solution *u*_*g*_ for genes *g* and the data $u_{g}^{dat}$ is summed over all genes and over *T* times at which the data is available; σ_*g, k*_ is the standard deviation of the data for gene *g* and time *t*_*k*_. For fits to the CDC Frontier data, *wRSS* was additionally complemented with a penalty term equal to the covariance between the model solution and data.

The model fitting was performed either to the LD data only (and the SD data was used for testing) or to the joint LD and SD data, in which case *wRSS* from equation (9) should be calculated for the two growth conditions and summed. In the case of the LD fits, there were 35 data points in total for ICCV and 75 data points for CDC Frontier. In the case of fits to the joint SD and LD data, there were 70 and 145 data points for ICCV and CDC Frontier, respectively. The expression data for the five genes under modeling and the five *FT* homologs in chickpea was obtained from Figure 5 of the paper by Ridge et al. (2017). The figure was digitized by the web-based tool WebPlotDigitizer (Rohatgi, 2018; the extracted expression data is available at https://zenodo.org, DOI:10.5281/zenodo.1451748). The cost functional was minimized by the differential evolution, which is a global parameter search method, using either a wolframscript program utilizing *NMinimize* function in Wolfram Mathematica or an entirely parallelized version of the method implemented in the DEEP software (Kozlov et al., 2016).

We assessed the quality of the alternative models *H0*–*H2* using the Akaike information criterion adjusted for small data samples:

(10) $$AICc=2k-2\text{log}\hat{L}+\frac{2k^{2}+2k}{m-k-1}\;,$$

where *k* is the number of parameters in a model, *m* is the number of data points used for model fitting, and $\hat{L}$ is the maximum value of the likelihood function. In our case, $2log\;\hat{L}=-wRSS_{\min}$ — the minimal value of the *wRSS* functional from equation (9) estimated from the set of model fits (see Supplementary Text for derivation of $\hat{L}$). We also used a classical likelihood function appearing in least squares fitting.

## Author contributions

MS and SN conceived and coordinated the project. VG and KK conducted the computational experiments. VG analyzed and summarized the results and wrote the first draft of the manuscript. All the authors participated in finalizing the manuscript.

### Conflict of interest statement

The authors declare that the research was conducted in the absence of any commercial or financial relationships that could be construed as a potential conflict of interest.
