# Supplementary material for: Dynamical Modeling of the Core Gene Network Controlling Flowering Suggests Cumulative Activation From the FLOWERING LOCUS T Gene Homologs in Chickpea
Source: Front Genet. 2018 Nov 20;9:547. doi: 10.3389/fgene.2018.00547 (PMC6262361; doi:10.3389/fgene.2018.00547)
Supplement: Supplementary file 1 [file Data_Sheet_1.PDF]

Supplementary Material

Dynamical Modeling of the Core Gene Network Controlling Flowering Suggests Cumulative Activation from the *FLOWERING LOCUS T* Gene Homologs in Chickpea

Vitaly V. Gursky*, Konstantin N. Kozlov, Sergey V. Nuzhdin, Maria G. Samsonova*

*** Correspondence:** Vitaly V. Gursky, gursky@math.ioffe.ru, and Maria G. Samsonova, m.g.samsonova@gmail.com

1 Supplementary Text: Maximum likelihood estimation under prescribed data variance

We consider a data sample $x = \{x_1, \ldots, x_n\}$, where x_i represents a protein concentration at a time t_i, and n is the total number of such data values. For each t_i, we have experimental estimate σ_i^2 of the variance of x_i. We assume that x_i are realizations of the random variables $X_i = h(t_i, \theta) + \varepsilon_i$, where $h(t, \theta)$ is the model solution with the vector of parameters θ, and the errors ε_i are random variables with some probability distribution. We assume $\varepsilon_i \sim N(0, \sigma_i^2)$ — normal distribution with zero mean and experimentally estimated variance σ_i^2. Therefore, this corresponds to the assumption that, at the true parameter set $\hat{\theta}$, the discrepancy between the model and the data only comes from the uncertainty of the data, quantified by the variances σ_i^2.

The likelihood function $\mathcal{L}(\theta|x)$ is then written as the probability density function for the sample of n independent non-identically distributed normal random variables:

$$\mathcal{L}(\theta|x_1, \ldots, x_n) = f(x_1, \ldots, x_n|\theta, \sigma_1^2, \ldots, \sigma_n^2) = \prod_{i=1}^{n} f(x_i|\theta, \sigma_i^2)$$

$$= \left(\prod_{i=1}^{n} \frac{1}{\sqrt{2\pi\sigma_i^2}} \right) \exp\left(-\sum_{i=1}^{n} \frac{(x_i - h(t_i, \theta))^2}{2\sigma_i^2} \right).$$

This leads to the following log-likelihood:

$$\log \mathcal{L}(\theta|x) = \text{const} - \sum_{i=1}^{n} \frac{\left(x_i - h(t_i, \theta)\right)^2}{2\sigma_i^2} = \text{const} - \frac{1}{2} wRSS,$$

where *wRSS* denotes the weighted residual sum of squares in this formula (presented in eq. (9) of Materials and Methods of the main text), and the constant term can be omitted under the

maximization. Finally, the maximal value $\hat{\mathcal{L}}$ of the likelihood function is derived from the minimal value of *wRSS*:

$$\log\hat{\mathcal{L}} = \max_{\theta} \log\mathcal{L}(\theta|x) = -\frac{1}{2}\min_{\theta} wRSS.$$

Therefore, the term $(-2\log\hat{\mathcal{L}})$ in the Akaike information criterion can be replaced by $wRSS_{\min}$.

In the classical least squares fitting, the error is assumed to be distributed independently and identically, i.e. $\sigma_i = \sigma$ for all i, and σ is an additional parameter (Kenneth and Burnham, 2003). The maximal log-likelihood in this case is expressed via the maximum likelihood estimate $\hat{\sigma}^2$ for this variance as follows (up to a constant):

$$\log\hat{\mathcal{L}} = -\frac{n}{2}\log\hat{\sigma}^2 = -\frac{n}{2}\log\frac{RSS}{n}, \qquad (S1)$$

where *RSS* is the residual sum of squares. Results of AICc comparison between models if this formula for the maximal log-likelihood is used are shown in Supplementary Figure 8.

2 Supplementary Figures

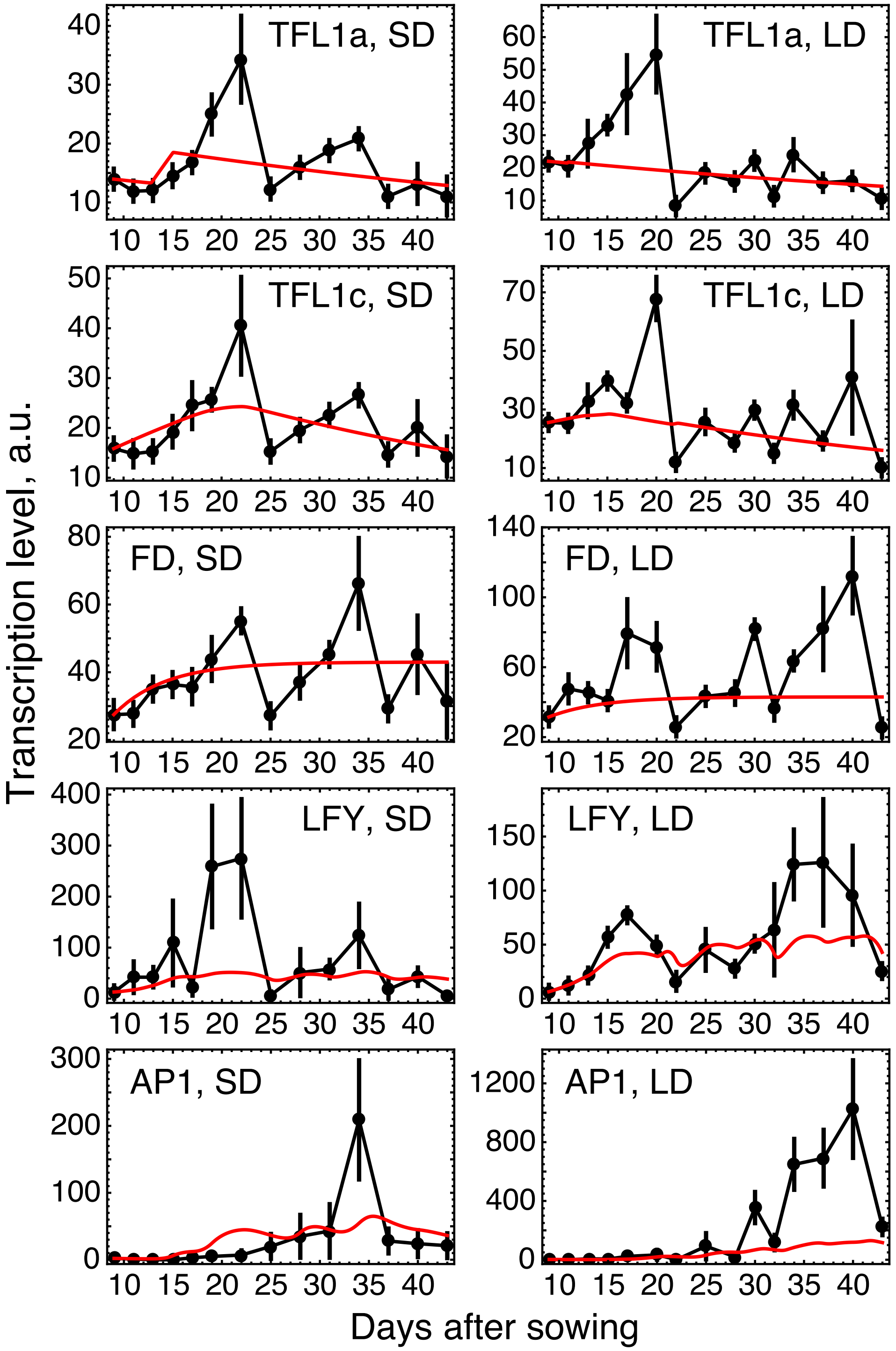

Supplementary Figure 1. The best solution (red curve) in model *H0* for CDC Frontier. Black dots and ranges are the means and s.d., respectively, of the expression values from the CDC Frontier data by (Ridge et al., 2017).

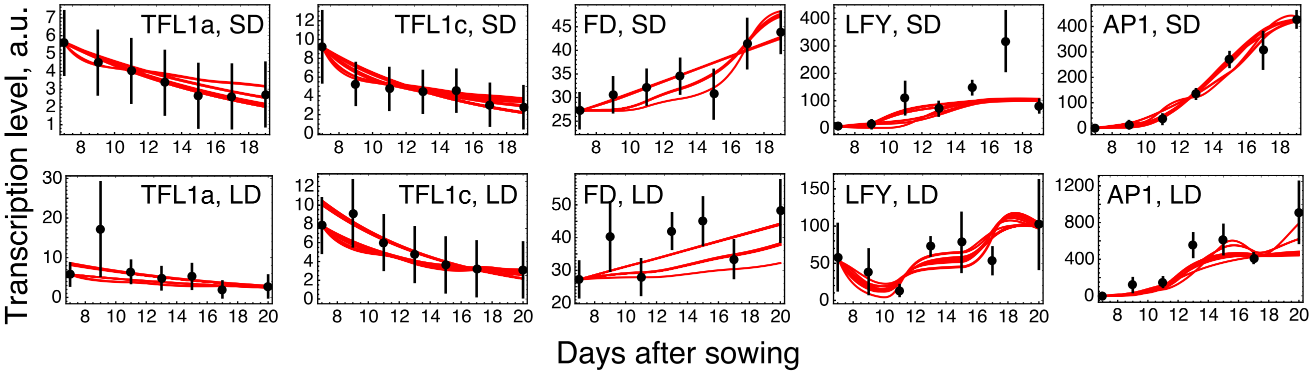

Supplementary Figure 2. Model *H0* solutions for ICCV 96029 obtained by model fitting to the LD data only. The model solutions (red curves) corresponding to all parameter sets found by optimization are shown for five flowering time genes and for the short day (SD, upper panels) and long day (LD, lower panels) conditions. The black dots and error ranges are the mean expression data and standard deviation, respectively, taken from (Ridge et al., 2017). The solutions exhibit the same characteristic features as the solutions obtained by model fitting to the joint SD+LD data, shown in **Figure 2** of the paper.

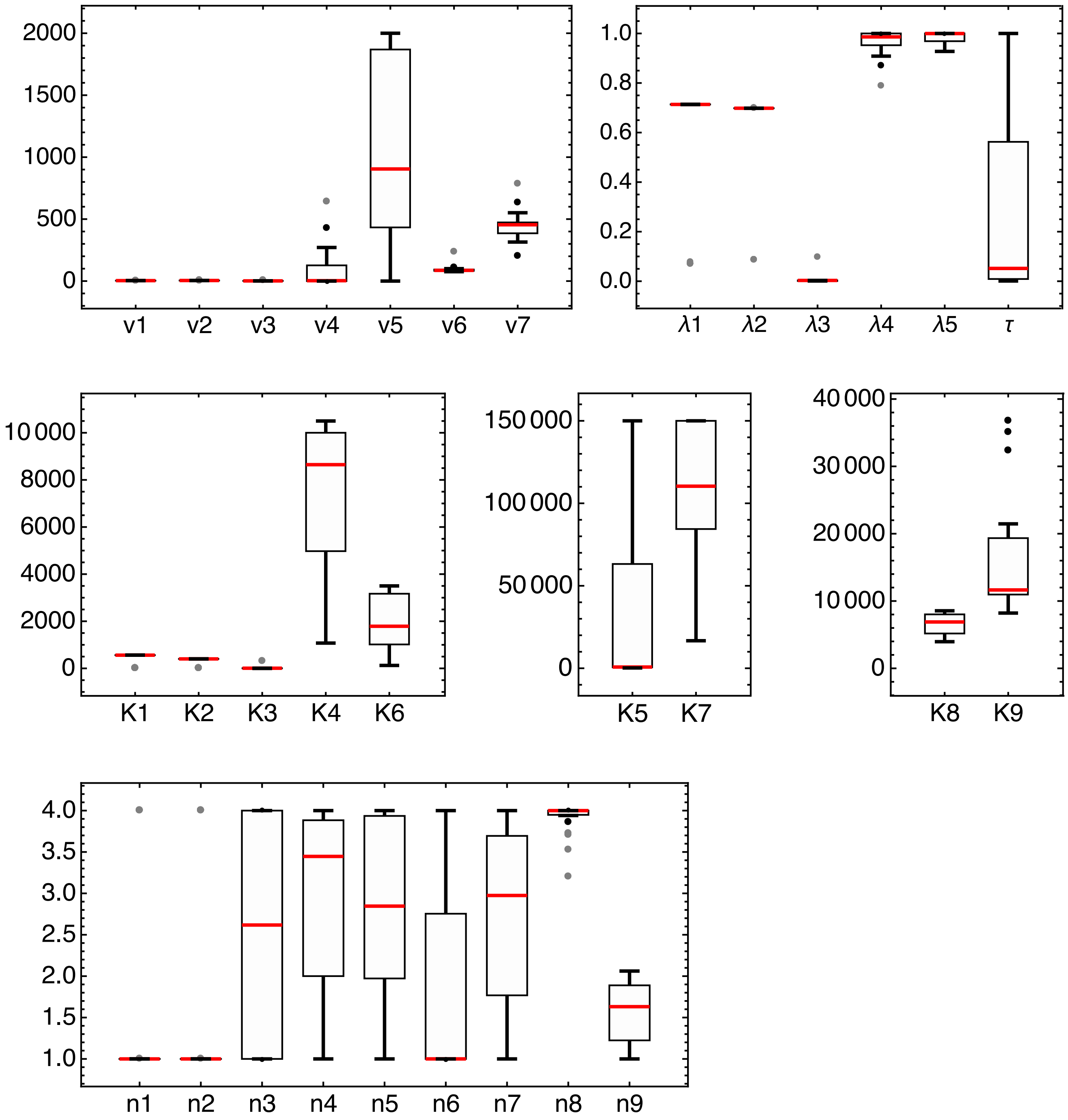

Supplementary Figure 3. Distributions of the estimated parameter values in model *H0*. The designations for parameters are as described in Materials and Methods of the main text (see eqs. (1)–(6)). The parameters λ_i and τ have distinct dimensionalities (sec.$^{-1}$ and sec., respectively), but are shown in the same panel for simplicity, because they have the same range in their units. The equilibrium dissociation constants K_5, K_7, K_8, and K_9 were separated from the rest of K_i's because they correspond to the regulation by the protein complexes FD-TFL1 (K_5 and K_7) and FD-FT (K_8 and K_9) and, thus, implicitly contain the constants (c_1 and c_2) of proportionality between the concentration of the complexes and the concentrations of the substrates: $[\text{FD-TFL1}] = c_1[\text{FD}]([\text{TFL1a}] + [\text{TFL1c}])$, $[\text{FD-FT}] = c_2[\text{FD}]([\text{FTa1}] + \cdots + [\text{FTc}])$.

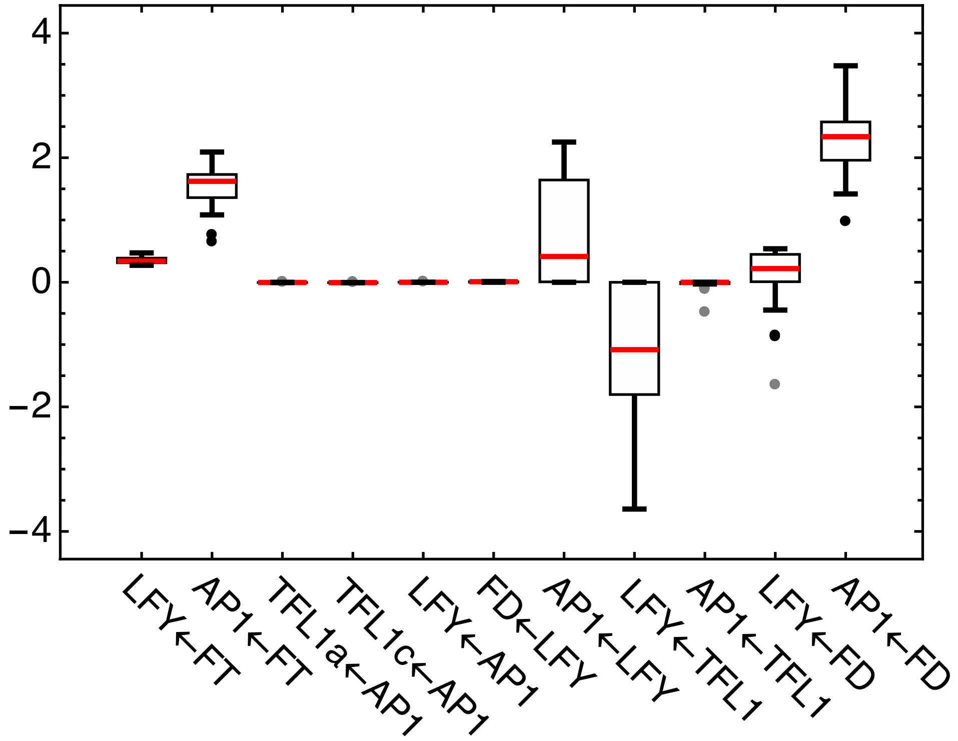

Supplementary Figure 4. Average values of the Jacobian of the right-hand side of the model equations for all sets of the optimized parameter values in model *H0* for ICCV 96029. The Jacobian term for a regulation $A \leftarrow B$ is the derivative of the right-hand side of the model equation for u_A w.r.t. the regulator concentration u_B. For each such regulation and each set of the optimized parameter values, the average value of such Jacobian term was obtained by integrating this term over time under SD and LD and dividing by the integration time interval; the figure shows box plots of the distributions of these values over all sets of the optimized parameter values. The type of regulation corresponding to each Jacobian term is shown on the horizontal axis, where arrow indicates the direction of the regulation. Dots show outliers. The Jacobian values for the *FT*-like genes as the regulators are equal to each other under the cumulative regulation (model *H0*), so this single value multiplied by 5 (the number of the *FT*-like genes) is shown in the figure. Since TFL1a and TFL1c appear in the model equations only as the sum, the Jacobian terms w.r.t. these proteins are also equal to each other; the averaged values corresponding to TFL1a only are shown for the cases in which TFL1 is the regulator.

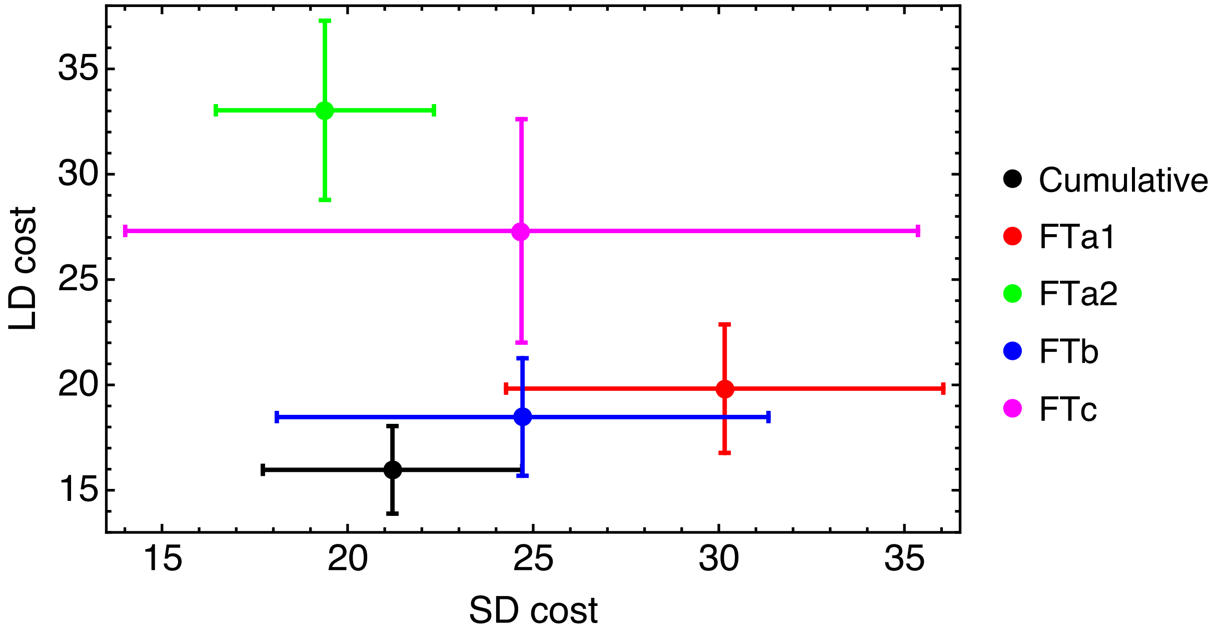

Supplementary Figure 5. The means and standard deviations of the SD- and LD-related costs in model *H0* (black) and four versions of model *H1* (colored), for ICCV 96029. The full cost function *wRSS* (eq. (9) in Materials and Methods of the main text) is the sum over all times in SD and LD growth conditions. The SD-related cost is obtained by taking only the times from SD in that sum, and similarly for the LD-related cost. The SD- and LD-related costs show the performance of each model only under SD and LD conditions, respectively. Each color corresponds to a version of model *H1* in which a single *FT* gene (indicated in the legend) taken as the only FT-activator in the model. All versions of model *H1* perform worse than *H0* in terms of the LD-cost, i.e. the differences in the LD-costs between model *H0* and all versions of *H1* are significant (p-value < 0.0002). In terms of the SD-cost, model *H1* with *FTa2* taken as the sole FT-activator performs slightly better than *H0* (p-value = 0.03). Model *H1* with *FTc* taken as the sole FT-activator performs as well as *H0*, i.e. the difference between these two SD-costs are not significant (p-value = 0.57). All other *H1* models perform worse than *H0* in terms of the SD-cost (p-value < 0.02).

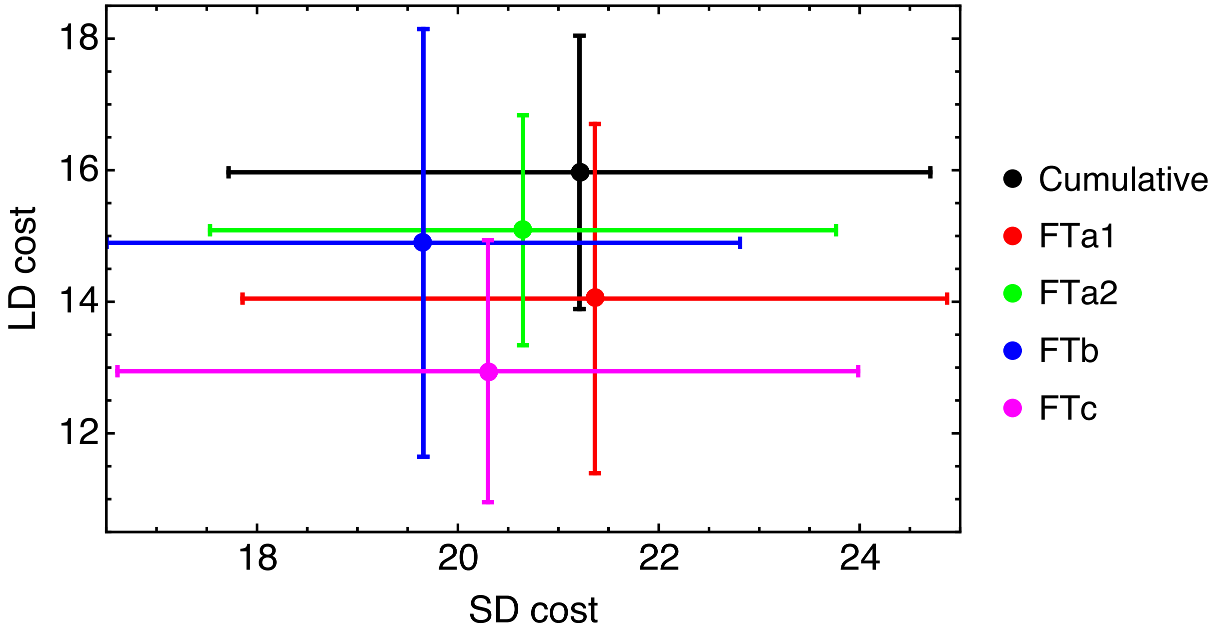

Supplementary Figure 6. The means and standard deviations of the SD- and LD-related costs in model *H0* (black) and four versions of model *H2* (colored), for ICCV 96029. The full cost function *wRSS* (eq. (9) in Materials and Methods of the main text) is the sum over all times in SD and LD growth conditions. The SD-related cost is obtained by taking only the times from SD in that sum, and similarly for the LD-related cost. The SD- and LD-related costs show the performance of each model only under SD and LD conditions, respectively. Each color corresponds to a version of model *H2* in which a single *FT* gene (indicated in the legend) is represented via a separate Hill function in the model equations, with all other *FT*-genes conferring the cumulative activation. All versions of model *H2* perform as well as *H0* in terms of the SD-cost, i.e. the differences in the SD-costs between model *H0* and all versions of *H2* are not significant (p-value > 0.14). In terms of the LD-cost, models *H2* with *FTa1* and *FTc* separated perform slightly better than *H0* (p-values = 0.004 and 7×10^{-6}, respectively).

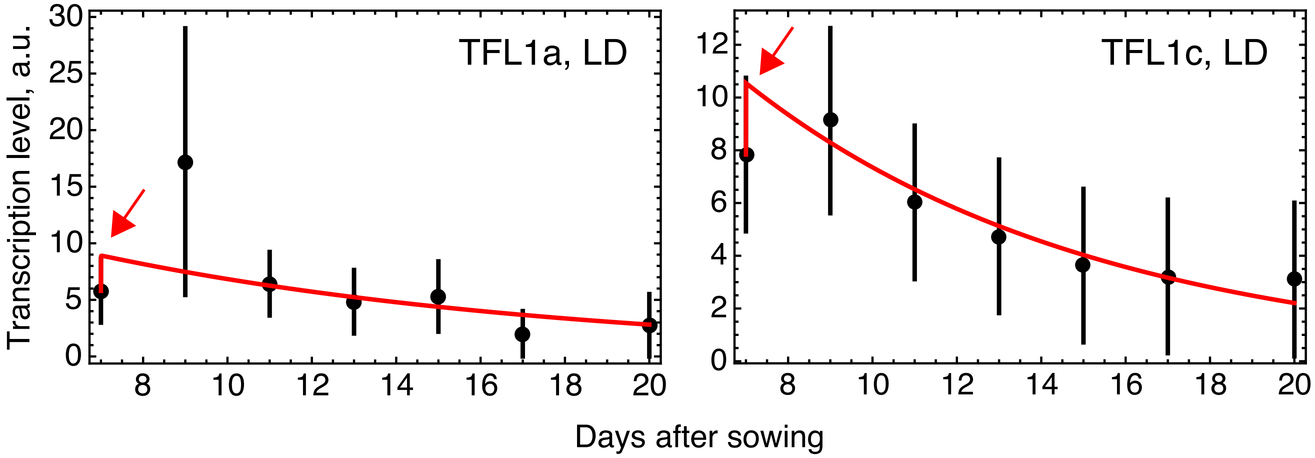

Supplementary Figure 7. Model solutions for TFL1a and TFL1c (red curves) at the best optimized parameter values exhibit undesirable behavior (indicated by the arrows) in LD, for ICCV 96029 data. Such artefacts appear if the initial conditions for TFL1a and TFL1c are set to the first data value (at *t* = 7 days after sowing). The solutions at the optimized parameter values tend to increase the concentrations of these proteins very rapidly at early times. This pushes the maximal expression rates under optimization to the upper limits for these proteins: $v_1 = v_2 = 10000$. To avoid this effect, the initial conditions for these proteins were set to zero at *t* = 0, and the functions in the right-hand side of the model equations were obtained by interpolating the data values back to zero concentrations at *t* = 0.

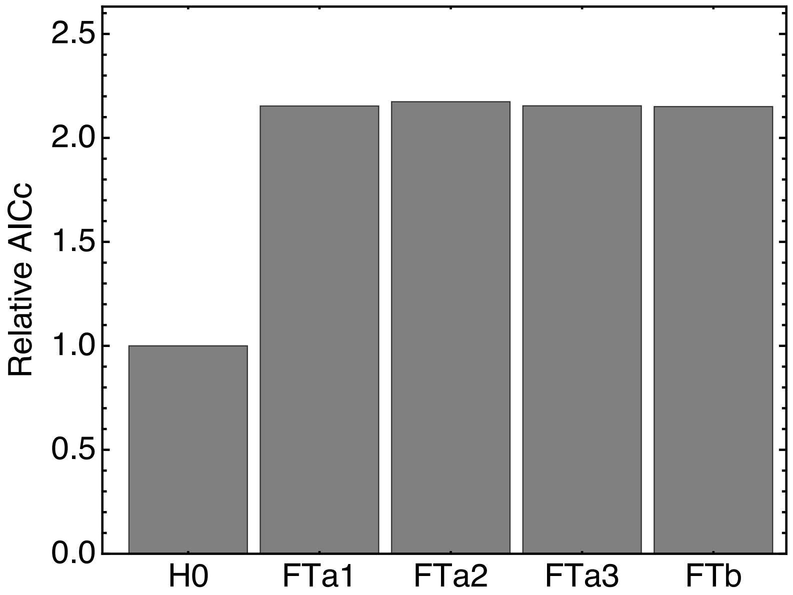

Supplementary Figure 8. Version of **Figure 6D** from the main text, but for the classical AICc formula used in least squares fitting. The expression from eq. (S1) from the Supplementary Text is used for $\log \hat{\mathcal{L}}$ in the AICc formula.

References

Kenneth, P., Burnham, D. A. (2002). *Model Selection and Multimodel Inference: A Practical Information-theoretic Approach* (2nd ed.). Springer. 515 pp. ISBN 0-387-95364-7.

Ridge, S., Deokar, A., Lee, R., Daba, K., Macknight, R. C., Weller, J. L., et al. (2017). The chickpea Early flowering 1 (Efl1) locus is an ortholog of arabidopsis ELF3. *Plant Physiol.* 175, 802–815. doi:10.1104/pp.17.00082.
